# Response to fever and utilization of standby emergency treatment (SBET) for malaria in travellers to Southeast Asia: a questionnaire-based cohort study

**DOI:** 10.1186/s12936-017-1678-2

**Published:** 2017-01-25

**Authors:** Christof D. Vinnemeier, Camilla Rothe, Benno Kreuels, Marylyn M. Addo, Sabine Vygen-Bonnet, Jakob P. Cramer, Thierry Rolling

**Affiliations:** 1 I. Department of Medicine, Section Tropical Medicine, University Medical Center Hamburg-Eppendorf, Hamburg, Germany; 2 Clinical Research Group, Bernhard Nocht Institute for Tropical Medicine, Hamburg, Germany; 3 Infectious Disease Epidemiology, Bernhard Nocht Institute for Tropical Medicine, Hamburg, Germany; 4 Department for Infectious Disease Epidemiology, Robert Koch Institute, Berlin, Germany

**Keywords:** Malaria, Standby emergency treatment, SBET, Southeast Asia, Travellers, Healthcare-seeking behaviour, Malaria guidelines, Pre-travel advice

## Abstract

**Background:**

Guidelines in several European countries recommend standby emergency treatment (SBET) for travellers to regions with low or medium malaria transmission instead of continuous chemoprophylaxis: travellers are advised to seek medical assistance within 24 h in case of fever and to self-administer SBET only if they are not able to consult a doctor within the time period specified. Data on healthcare-seeking behaviour of febrile travellers and utilization of SBET is however scarce as only two studies were performed in the mid-1990s. Since tourism is constantly increasing and malaria epidemiology has dramatically changed in the meantime more knowledge is urgently needed.

**Methods:**

Some 876 travellers to destinations in South and Southeast Asia with low or medium malaria transmission were recruited in the travel clinic of the University Medical Center Hamburg-Eppendorf. Demographic and travel-related data were collected by using questionnaires. Pre-travel advice was carried out and SBET was prescribed in accordance to national guidelines. Post-travel phone interviews were performed to assess health incidents during travel and individual responses of travellers to febrile illness.

**Results:**

Out of 714 patients who were monitored, 130 (18%) reported onset of fever during travel or 14 days after return. Of those travellers who reported fever, 100 (80%) carried SBET during travel. The vast majority of 79 (79%) febrile travellers who carried SBET did not seek medical assistance. Overall, 14 (14%) febrile patients who carried SBET and six (20%) patients who did not carry SBET took the correct measure (doctor visit or timely SBET administration) as a response to febrile illness, respectively. Only two travellers self-administered SBET, but both of them applied the wrong regimen.

**Conclusions:**

In view of declining malaria transmission and improving medical infrastructure in most countries of Southeast Asia and obvious obstacles concerning SBET as shown in this study the current strategy should be re-evaluated. Pre-travel advice concerning malaria in SEA should focus on appropriate mosquito bite protection and clearly emphasize the need to see a doctor within 24 h after onset of fever.

## Background

National recommendations for malaria prophylaxis for travellers to low- or intermediate-risk areas vary. Depending on seasonality, mode of travel, length of stay, travellers are advised to perform mosquito protection and chemoprophylaxis or carry standby emergency treatment (SBET). SBET is a concept according to which travellers carry anti-malarials (e.g., atovaquone/proguanil or artemether/lumefantrine), which have to be self-administered in case of fever and the inability to rule out malaria as the cause of fever within 24 h. The rationale behind the strategy of SBET is that the potential occurrence of side effects during continuous chemoprophylaxis might outweigh the relatively small risk of developing malaria in areas of low malaria transmission. The continuous decrease in malaria incidence in many low-transmission areas, e.g., in South- and Southeast Asia (SEA), as well as increasing drug resistance have also served as arguments in favour of SBET. The Societies for Tropical Medicine of Germany and Switzerland, for example, promote SBET for all areas of low and medium malaria transmission along with protection against mosquito bites instead of chemoprophylaxis [1]. The World Health Organization (WHO) recommends this approach to travellers “in some occupational groups who make frequent short stops in countries or areas with malaria risk over a prolonged period of time” and short-term travellers to “remote rural areas where there is very low risk of infection” [2]. The Centers for Disease Control and Prevention (CDC) in the USA, in contrast, recommend to carry SBET to ensure high-quality treatment in case of a diagnosis of malaria, accounting for the increasing numbers of fake anti-malarials in some countries of SEA [3].

While the concept of SBET appears straightforward in theory, it is unclear whether or not travellers are willing or able to apply SBET correctly while being abroad. In fact, there has been repeated criticism that travellers may be unable to make correct decisions in case of onset of fever during travel and tend to apply medication incorrectly [4, 5]. To date only two publications from 1995 are available concerning these issues. Both studies demonstrated that only a small fraction of travellers had to self-administer SBET. Additionally, when *Plasmodium falciparum* antibody levels were assessed in all travellers after return it turned out that even this low number of self-administrations proved to be a massive overuse of SBET, since most SBET users had no antibodies [4, 6].

Moreover, the SBET concept is based on the assumption that reliable medical care is generally not available at malaria-endemic tourist destinations. Yet, infrastructure for tourism, as well as medical emergency care has improved significantly in the past decades in many countries of SEA. At the same time, numbers of travellers to those regions have increased substantially due to a major surge in the availability of flight connections as well as a dramatic decrease in airline fares [7]. Concurrently to increased travel, malaria transmission in most parts of SEA has decreased, resulting in smaller numbers of imported malaria from the region [8, 9]. India also recorded a decline in malaria cases, but predominantly through control of falciparum malaria. In contrast, the relative proportion of *Plasmodium vivax* cases was increasing on the Indian sub-continent [10].

The aim of the present study was to assess utilization of SBET in the face of changing background conditions. Therefore, a cohort study in a population travelling to areas of low and medium malaria transmission in South Asia and SEA was performed.

## Methods

### Study design and recruitment

Between October 2013 and November 2014, a prospective, questionnaire-based cohort study was conducted at the travel clinic of the University Medical Center Hamburg-Eppendorf. Travellers aged ≥18 years with intention to travel to South Asia and SEA were screened for eligibility in the waiting area prior to seeing a trained physician for travel medicine advice. The following destination countries were defined for participation: India, Thailand, Laos, Cambodia, Vietnam, Sri Lanka, Myanmar, Indonesia, Bangladesh, the Philippines, or Malaysia. Additional inclusion criteria were travel duration between 7 days and 12 weeks and willingness to provide contact data for a post-travel interview as well as signed informed consent to participate. Study procedures comprised completion of a pre- and post-travel questionnaire.

#### Pre-travel interview

The pre-travel questionnaire had to be completed before travel medicine consultation with a physician. The pre-travel questionnaire comprised questions concerning the travel destination, mode of travel and basic health data of the participant, such as pre-existing illness and regular medication. To avoid a possible behavioural bias of study participants regarding the awareness of fever or application of SBET, participants were informed that a post-travel telephone interview would be conducted concerning health incidents during and after travel without specifying precise question items or topics.

Standby emergency treatment was prescribed according to the current recommendations of the German Society of Tropical Medicine (DTG) for the years 2013/2014 [1]. In brief, the DTG advised travellers to all countries, except certain regions in Indonesia, to carry out mosquito bite prevention at all times and to carry SBET, such as atovaquone/proguanil or artemether/lumefantrine. For regions in Indonesia located east of Bali, travellers were advised to take continuous chemoprophylaxis either with atovaquone/proguanil or mefloquine.

Use of SBET was thoroughly explained, travellers were advised to take a thermometer with them and administer SBET only in case of fever with temperatures of >38 °C and if they were unable to seek medical advice within 24 h. Fever, chills, myalgia, and symptoms suggestive of a common cold were mentioned as key symptoms to prompt travellers to seek medical advice. Additionally, a leaflet with all instructions was handed out to all participants. Participation in the study did not influence recommendations of preventive measures against mosquito bites, vaccinations or prescription of SBET.

#### Post-travel interview

Between 4 and 6 weeks after travellers had returned home, all participants were contacted by a trained member of the study team to be screened for participation in the post-travel interview by asking the following four questions:

1. Did you experience fever, chills or flu-like symptoms during your travelling abroad or within 14 days after returning home?
2. Did you consult a doctor during your travelling abroad or within 14 days after returning home?
3. Did you or somebody from your travel group carry any anti-malarial medication (e.g., mefloquine, atovaquone/proguanil, doxycycline, artemether/lumefantrine) or buy one in the destination country?
4. Did you self-administer any anti-malarial medication?

If one of these questions was answered with “Yes”, travellers qualified for participation in the detailed post-travel questionnaire concerning symptoms, date and place of onset of symptoms, doctor visit, diagnosis and medication.

### Data analysis

Data analysis was carried out by using Stata v11.1 (StataCorp, College Station, TX, USA). Data analysis was descriptive and no statistical hypothesis was tested.

### Notification data on malaria

To define the context in which standby emergency treatment is currently used in Germany, notification data of malaria cases from the destination countries covered by the study were analysed. In Germany, notification of malaria cases by laboratories is mandatory and based on the direct detection of the malaria parasite in the human blood. The diagnosing laboratory reports directly to the Robert Koch Institute (RKI). A second data form with information on travel destinations and purposes, clinical findings, prophylaxis, and treatment is completed by the attending physician. The department of infectious disease epidemiology of the RKI joins the information of both data forms in a unique database and analyse the data on an annual basis. Despite the mandatory nature, notification data are incomplete: not all cases are reported; for some cases the RKI receives only one of the two data forms; some of the data forms are only partially filled out.

### Ethical considerations

The study protocol was approved by the ethics committee of the Medical Council in Hamburg, Germany. Prior to recruitment and pre-travel advice, eligible travellers were informed about the study by a member of the study team. They were included only after providing written informed consent.

## Results

### Characteristics of travellers and itineraries

Out of 1671 travellers screened for eligibility, pre-travel questionnaires were completed by 876 travellers with a median age of 32 years [interquartile range (IQR) 17–45]. Gender distribution was about equal (52.7% women). The majority of participants were well educated, more than half (53.9%) of them held a university degree. Pre-existing illness was reported by only a minor proportion of 14.6% of study participants. Thyroid disease and chronic respiratory diseases accounted for the most frequent disorders with 2.5 and 2.1%, respectively. Cardiac disease and diabetes were stated by eight (0.9%) and five (0.6%) of travellers. More than a quarter of travellers reported carriage of regular medication such as contraceptives (8.6%), drugs related to thyroid disease (4.9%) or hypertension (2.8%) (Table 1).

**Table 1:** Characteristics of travellers to Southeast Asia (n = 876)

Travellers’ characteristics		IQR
Age (median)	32	18 (27–45)

Travellers’ characteristics	n	%
Sex		
Male	414	47.3
Female	462	52.7
Education		
Tertiary degree	515	58.8
Upper secondary degree	212	24.2
Lower secondary degree	133	15.2
Primary	16	1.8
Pre-existing illness		
Any	128	14.6
Thyroid disease	22	2.5
Chronic respiratory diseases	18	2.1
Allergies	13	1.5
Hypertension	11	1.3
Neurologic disorders	11	1.3
Cardiac diseases	8	0.9
Diabetes mellitus	5	0.6
Other	61	6.9
Medication		
Any	260	26.3
Other	95	10.8
Contraceptive	75	8.6
Thyroid medication	43	4.9
Antihypertensives	25	2.8
Antiplatelet drugs	12	1.4
Drugs for respiratory diseases	10	1.1

*IQR* interquartile range

Thailand (35.6%), Vietnam (25.5%) and Cambodia (20.8%) were the most popular destinations. The median duration of travel was 21 days (IQR: 21–28). Top reason for travel was tourism, almost three-quarters of travellers intended to travel independently, i.e., without any kind of guidance. Only 32.4% of study participants had never travelled to (sub-) tropical regions before (Table 2).

**Table 2:** Itinerary characteristics (n = 876)

Itinerary characteristics		IQR
Duration of travel (days)	21	7 (21–28)

Itinerary characteristics	n	%
Destination		
Thailand	312	35.6
Vietnam	223	25.5
Cambodia	182	20.8
India	166	18.9
Indonesia	150	17.1
Malaysia	93	10.6
Laos	87	9.9
Sri Lanka	74	8.4
Myanmar	53	6.1
Philippines	48	5.5
Reason for travel		
Tourism	796	91.5
Business	29	3.3
Volunteering/education	30	3.4
Visiting friends and relatives (VFR)	15	1.7
Other	6	0.7
Type of travel		
Backpacking	340	38.8
Individual travelling	306	34.9
Organized round trip	120	13.7
Package holiday	26	3.0
Ship cruise	14	1.6
No answer	70	8.0
Previous travel experience		
1–2 times	285	32.5
3 to −5 times	169	19.3
>5 times	134	15.3
None	284	32.4
No answer	4	0.5

*IQR* interquartile range

### Post-travel

Of the 876 travellers recruited before travel, 714 could be contacted via telephone for a post-travel interview. Amongst these, 130 (18.2%) reported onset of fever during travel or within 14 days after return. Of these, 31.5% reported concomitant diarrhoea, 14.6% reported vomiting. Myalgia, chills and other flu-like symptoms were reported by 47.7% (Table 3).

**Table 3:** Symptoms of travellers reporting fever during travel or 14 days after return (n = 130)

Fever	n	%
Yes	130	18.2
Uring their travel	89	68.5
Ithin 14 days after return	41	31.5
No	559	78.3
No answer	25	3.5

Additional symptoms in febrile patients (n = 130)	n	%^a^
Diarrhoea	41	31.5
Myalgia	31	23.8
Chills	27	20.8
Headache	21	16.1
Vomiting	19	14.6
Sore throat	17	13.1
Abdominal pain	15	11.5
Tiredness	12	9.2
Stomache ache	8	6.2
Flu-like symptoms	4	3.1
Vertigo	3	2.3
Symptoms of sinusitis	3	2.3

^a^May not sum up to 100% since some patients recalled multiple symptoms

### SBET utilization

**Fig. 1:**
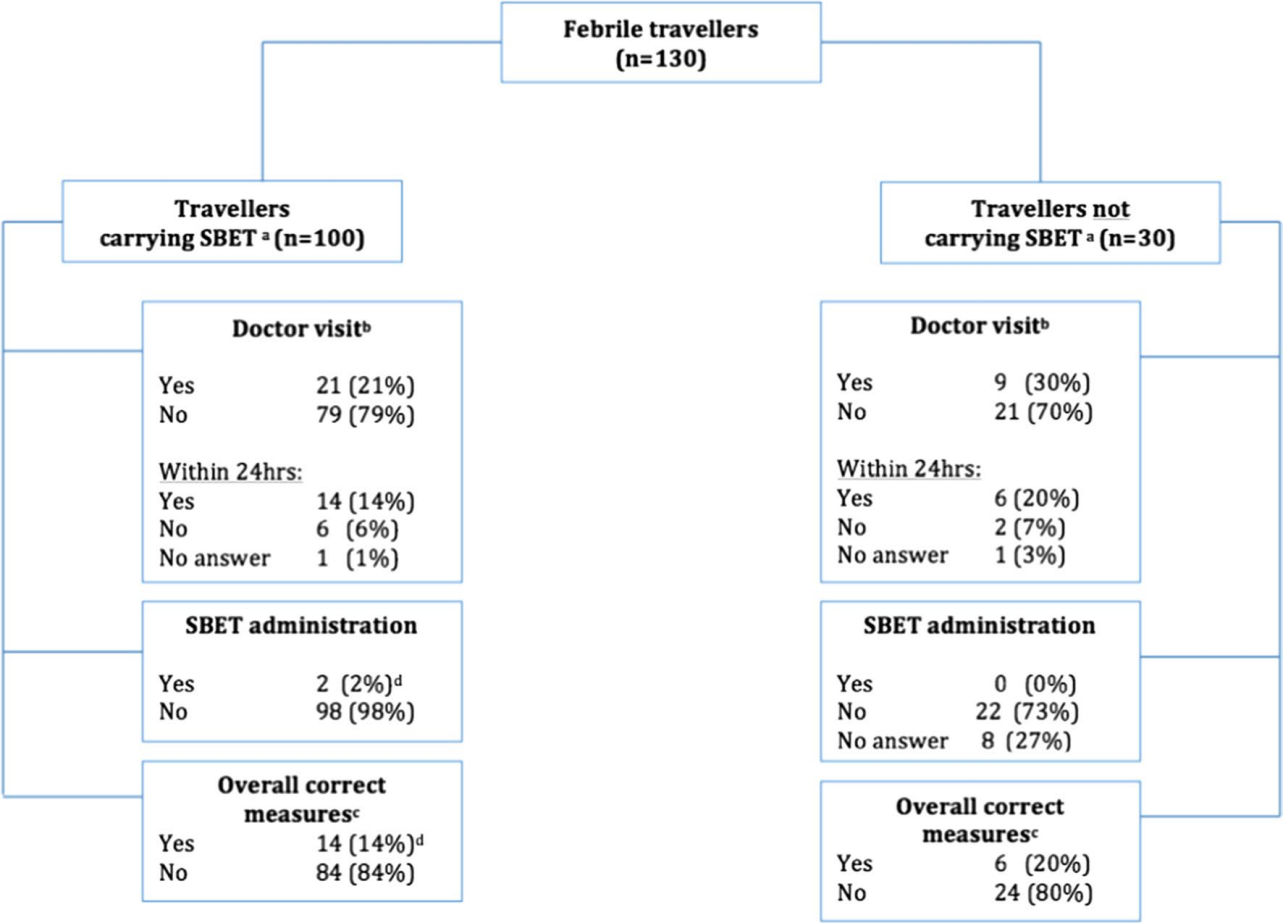
Responses of travellers to febrile illness. ^a^Stand-by emergency treatment. ^b^Doctor visit in country of travel. ^c^Correct measures: febrile travellers took correct measures if they sought for medical assistance within 24 h or self-administered SBET. ^d^Since both travellers applied an incorrect scheme of SBET, administration of the medication was not counted as “correct measure”

Overall, 511 (71.6%) travellers carried SBET during travel. Out of 130 febrile travellers, 100 (76.9%) carried SBET during travel. Amongst those febrile travellers who carried SBET, 21 (21%) attended a local medical care facility because of the fever, only 14 (14%) of those within 24 h after onset of fever. Only 2 out of 714 travellers self-administered SBET in terms of emergency medication, but did not apply the correct scheme (Fig. 1). A 25-year old male traveller to Malaysia and Indonesia stated to have mistakenly applied one tablet of atovaquone/proguanil during his stay in Malaysia due to “lack of knowledge”. After his return to Germany he experienced fever, chills and diarrhoea and consulted a doctor who diagnosed an infection with *Salmonella* spp.

Another 45-year old traveller to India experienced fever, diarrhoea and flu-like symptoms on his 21st day of travel. He did not attend a medical facility, but self-administered one tablet of atovaquone/proguanil seven days after onset of the symptoms, while he was staying in Kolkata/India (Table 4).

**Table 4:** Febrile travellers self-administering SBET (n = 2)

Sex, age (years)	Destination	Complaints	Consultation of doctor	Place of SBET administration	Correct regimen
Male, 25	Malaysia, Indonesia	Fever, chills, diarrhoea; onset after return to Germany	In Germany	Malaysia	No
Male, 45	India	Fever, diarrhoea, flu-like symptoms; onset travel day 21	No	Kolkata, India	No

Out of 30 febrile travellers not carrying SBET during travel, nine (30%) visited a healthcare facility after the onset of fever, six of those within 24 h. Overall, 14 travellers (14%) experiencing fever during travel followed instructions as recommended by pre-travel advice by carrying SBET and visiting a health care facility within 24 h. None of the 14 travellers visiting a clinic for fever, took SBET, since malaria was ruled out at the health facility. Among travellers who did not experience fever during travel, no one self-administered SBET as emergency medication.

### Recorded cases of imported malaria from South Asia and SEA

Table 5 shows the distribution of notified imported malaria cases from South Asia and SEA to Germany between 2011 and 2015. Throughout this period, most travellers with malaria returned from India and Indonesia, while no malaria was imported from Myanmar and Laos. The majority of notified cases was attributable to *P. vivax* infections (46.6%), while only 13 (17%) cases of *P. falciparum* were notified.

**Table 5:** Reported cases of malaria imported into Germany from SEA between 2011 and 2014

	2011	2012	2013	2014	2015	Total
Country of travel						
Myanmar	0	0	0	0	0	0
India	17	17	1	5	5	45
Indonesia	4	1	1	1	2	9
Cambodia	1	0	0	1	2	4
Laos	0	0	0	0	0	0
Malaysia	0	0	0	0	1	1
Philippines	0	0	0	0	1	1
Sri Lanka	0	0	1	0	0	1
Thailand	0	1	3	1	2	7
Vietnam	0	0	0	1	0	1
Not specified	2	5	1	0	0	8
*Plasmodium* species						
Total (all species)	24	24	7	9	13	77
*Plasmodium falciparum*	0	5	1	3	4	13
*Plasmodium vivax*	18	15	4	4	5	46
Other and non-specified	1	1	2	1	1	6

Cases reported to the Robert Koch Institute Berlin, Germany

## Discussion

A substantial proportion of 18% of travellers to regions of South Asia and SEA with medium and low risk for malaria transmission reported febrile illness during travel. Only very few travellers adhered to the pre-travel advice to seek medical support within 24 h in case of fever. Only 2 out of 714 travellers self-administered SBET during travel, but both of them applied an incorrect regimen and took a single tablet only, which would not have any therapeutic effect in case of true malaria. The study team is not aware of any case of malaria in the study population.

In assessing the concept of SBET, three main problems were identified in travellers with fever during travel: (i) a major proportion of travellers did not carry SBET although it was prescribed; (ii) non-adherence to pre-travel advice while being abroad; and, (iii) incorrect self-administration of SBET.

Travellers recruited for this study were young with a median age of 32 years, healthy, disproportionally well educated and the majority was proficient with travelling to (sub-) tropical countries. In this respect characteristics of this population are comparable to those from other studies and it seems justifiable to assume, that the current results can be generalized to other traveller populations attending pre-travel clinics [11, 12].

Despite the changed landscape of malaria since 1995 when the last data on SBET utilization was published, the findings are in line with the prior studies. In a Swiss study from 1995, only six (0.5%) out of 123 febrile travellers applied SBET and only four of them applied the correct regimen [4]. In a German multi-centre study, 1.4% (40/2867) of travellers reported SBET use. Malaria antibody levels were later demonstrated in four participants who applied SBET [6]. Increasing evidence therefore indicates that only a small proportion of travellers to low risk malaria areas adheres to pre-travel advice based on the SBET concept.

The low acceptance and carriage rate of SBET in this study population is however surprising. Only 16% of travellers carrying SBET took any correct measure and 20% of travellers not carrying SBET sought medical assistance after the onset of fever which is a strikingly low figure regarding the standardized pre-travel advice and the educational level of the study population. Explanations for this result may be various: available data suggest that the recall rate of information after medical consultations showed overall good results suggesting that key messages seem to be well captured [13–15]. Since most consultations incorporate one or more vaccinations, injection anxiety, which has been shown quite common in some populations could be a potential distractor [16, 17]. However, the only available study in this context showed no association between recall of information and injection anxiety [18]. In any case, provision of simplified key messages after vaccination may facilitate better recognition of information. In general, studies assessing a traveller’s knowledge about travel-related health issues underlined a general increase of knowledge after pre-travel advice, so that other factors are likely to have contributed to non-adherence to emergency measures in case of fever in the current study [19–21]. In particular, in areas where population density and accessibility to medical facilities is good, tourists might tend to delay the decision to consult a doctor in favour of waiting for spontaneous recovery. Carriage of SBET could further encourage travellers to defer a medical consultation thereby waiting for the fever to drop. Challenges in adherence are not limited to the concept of SBET but are also seen for continuous malaria chemoprophylaxis for travellers to high-risk countries [22–26]. Several studies prove that the majority of returning travellers with malaria did not take malaria chemoprophylaxis or applied an incorrect regimen, the majority being travellers visiting friends or relatives (VFR) abroad [22, 27–31].

In consequence of an expanding tourism industry, travel to SEA is steadily increasing. For 2014, international tourist arrivals in the region rose to 96.7 million, which is a nearly fivefold increase since 1990 [7]. Concurrently to increasing travel, malaria transmission in South Asia and SEA has decreased over the past years. Between 2000 and 2015 the estimated number of malaria cases and deaths for the whole WHO region Southeast Asia declined by 39 and 37%, respectively [32]. It has been argued before that the malaria risk for travellers is not correlated with infection rates in the local population and attack rates in visitors may likely be higher because of the absence of partial immunity in contrast to the local populations [33]. Recent studies confirmed the trend of decreasing cases of imported malaria cases from SEA [9, 34]. According to data from malaria surveillance reports from the USA and 12 European countries, malaria cases imported from countries of SEA declined by 47% between 2003 and 2008 [8]. Another assessment of 320 imported cases of malaria between 1994 and 2012 from Denmark showed an annual decline of 6.5% [35].

However, India and Indonesia, as it is the case with notification data in Table 5, constitute two of the main source countries for imported malaria. In a retrospective analysis of national notification data from Canada, 9.2% of imported malaria cases originated from India. India displayed the most common source country for vivax malaria cases [36]. Whilst this should be taken into account during pre-travel advice, the notification data to the RKI presented in this study show low and declining numbers of malaria cases imported from South and SEA to Germany. Indeed, these data underline the economical impact of over-prescription of SBET, if compared to the annual numbers of travellers to SEA. Using figures provided by the German Travel Association (DRV), 1,769,825 individuals travelled to countries considered in this study in 2015 [37]. Considering a price of 42 Euro per unit, this would translate to 71.4 million Euros spent for SBET in 2015 in Germany alone. Facing the low number of imported falciparum malaria (13 cases during the past 5 years), these expenses hardly pass any cost-benefit analysis. In the light of the decreased numbers of imported malaria and the fact that the majority of reported cases are *P. vivax* cases, strategies for travellers to these regions have to be reassessed, in particular, because relapse of *P. vivax* is not adequately prevented by regular chemoprophylaxis or treated by a standard regimen of standby emergency treatment.

Even though atovaquone/proguanil is generally considered safe, up to 82% of patients reported an adverse event [38, 39]. The benefit-risk ratio for continuous malaria chemoprophylaxis is consecutively very low for most regions in SEA, and does not pose a viable alternative [40]. However, data from the current study reinforce the assumption that SBET has to be critically assessed. Equipping travellers with an anti-malarial has the potential to lead to a false sense of security and an uncritical perception of the risk of malaria during travel, favouring short-sleeved clothes and avoidance of repellents. Unattended administration of SBET harbours the risk of missing other medical conditions. The vast majority of travellers sojourn on popular tourist routes and visit similar places at their destinations. During the past two decades healthcare systems in most countries of South Asia and SEA have improved, although they remain at a low standard. However, from the main tourist tracks medical facilities can usually be reached within 24 h, particularly in metropolitan areas. One of the male travellers in this study who used SBET started intake of medication in Kolkata, where it would easily have been possible to find a hospital to rule out malaria [21]. There are however arguments in favour of SBET. First, travellers have advanced to more remote areas worldwide and carriage of SBET can be life-saving in cases when travellers are unable to reach a health facility due to missing infrastructure or external factors, e.g., severe weather conditions. Secondly, carriage of high-quality medication ensures safe treatment in view of increasing numbers of fake anti-malarials, especially in Asia [41–43]. Third, studies have shown that long-term travel can result in a higher cumulative risk for malaria [44, 45]. Those travellers may benefit from provision of SBET given the assumption that they tend to visit more rural regions. However, it is not possible to support this recommendation with the current study data, since only individuals travelling for no longer than 12 weeks were recruited.

This study has some limitations to be taken into account. As described above, a selective population of travellers visiting a health facility for pre-travel advice was studied. This population may not be comparable to the overall population travelling to South Asia and SEA. Earlier data demonstrated that travellers who attended a travel clinic have better knowledge about potential risks and protective measures [46]. Yet unpublished data from the Hamburg Airport Survey also suggest that travellers in the overall population have less frequently attended a travel clinic and are less frequently carrying SBET. Finally, it was not possible to completely rule out malaria in returned travellers, since no serology for *Plasmodium* spp. was performed. However, conduct of phone interviews 4–6 weeks after return makes unrecognized episodes of clinically relevant malaria unlikely.

## Conclusions

Only a very small proportion of travellers to low-risk malaria areas experiencing fever while abroad adhered to pre-travel advice related to the concept of SBET. Travel advice concerning malaria in South Asia and SEA should focus on appropriate mosquito bite protection and clearly emphasize the need to see a doctor within 24 h after onset of fever. Travellers with need of SBET should be carefully selected. Recommendations related to SBET should be revisited and limited to selected situations only, e.g., long-term travel or travel to remote rural areas.
